# Methylseleninic Acid Sensitizes Ovarian Cancer Cells to T-Cell Mediated Killing by Decreasing PDL1 and VEGF Levels

**DOI:** 10.3389/fonc.2018.00407

**Published:** 2018-09-28

**Authors:** Deepika Nair, Emelie Rådestad, Prajakta Khalkar, Nuria Diaz-Argelich, Axel Schröder, Charlotte Klynning, Johanna Ungerstedt, Michael Uhlin, Aristi P. Fernandes

**Affiliations:** ^1^Department of Medicine Huddinge, Center for Hematology and Regenerative Medicine, Karolinska Institutet, Stockholm, Sweden; ^2^Division of Biochemistry, Department of Medical Biochemistry and Biophysics, Karolinska Institutet, Stockholm, Sweden; ^3^Department of Clinical Science, Intervention and Technology, Karolinska Institutet, Stockholm, Sweden; ^4^Department of Organic and Pharmaceutical Chemistry, University of Navarra, Pamplona, Spain; ^5^Department of Gynecological Oncology, Karolinska University Hospital, Stockholm, Sweden; ^6^Hematology Center, Karolinska University Hospital, Stockholm, Sweden; ^7^Department of Applied Physics, Royal Institute of Technology, Stockholm, Sweden; ^8^Department of Clinical Immunology and Transfusion Medicine, Karolinska University Hospital, Stockholm, Sweden

**Keywords:** selenium, methylseleninic acid, PDL1, VEGF, HIF-1α

## Abstract

Redox active selenium (Se) compounds at sub toxic doses act as pro-oxidants with cytotoxic effects on tumor cells and are promising future chemotherapeutic agents. However, little is known about how Se compounds affect immune cells in the tumor microenvironment. We demonstrate that the inorganic Se compound selenite and the organic methylseleninic acid (MSA) do not, despite their pro-oxidant function, influence the viability of immune cells, at doses that gives cytotoxic effects in ovarian cancer cell lines. Treatment of the ovarian cancer cell line A2780 with selenite and MSA increases NK cell mediated lysis, and enhances the cytolytic activity of T cells. Increased T cell function was observed after incubation of T cells in preconditioned media from tumor cells treated with MSA, an effect that was coupled to decreased levels of PDL1, HIF-1α, and VEGF. In conclusion, redox active selenium compounds do not kill or inactivate immune cells at doses required for anti-cancer treatment, and we demonstrate that MSA enhances T cell-mediated tumor cell killing via PDL1 and VEGF inhibition.

## Introduction

Cancer cells are known to have an aberrant metabolism that gives rise to increased production of endogenous reactive oxygen species (ROS). In order to cope with augmented stress caused by the increased ROS, cancer cells maximize their antioxidant capacity rendering them more vulnerable to additional ROS levels. Therefore, increasing ROS levels through redox modulation may be a therapeutic strategy to selectively kill cancer cells but not normal cells (1). Selenium (Se) has been demonstrated to possess anti-cancer activity in several clinical and experimental studies (2), and one of the most well-studied mechanisms by which Se targets cancer cells is by inducing the production of ROS.

Se compounds have been shown to be selectively toxic to malignant cells, particularly to chemotherapeutic drug resistant cells, compared to normal cells (3). They exert their biological activity via their redox active metabolites. Two well characterized and widely used Se compounds are methylseleninic acid (MSA; CH_3_SeO_2_H) and sodium selenite (Na_2_SeO_3_) (2). Selenite is readily reduced by thiols to the key metabolite selenide that can redox cycle with oxygen and generate superoxide (4, 5). MSA is metabolized to methylselenol (CH_3_SeH) that is another important, redox active and central Se metabolite (1). Both compounds have shown anticancer properties, but with distinct modes.

Very little is known about the mechanistic effects of Se on the immune system, however Se compounds have been shown to influence the immune response by enhancing phagocytosis and inducing cytokine production (6). Treatment of tumor cells with selenite has been shown to result in loss of HLA-E expression thereby increasing susceptibility to CD94/NKG2A-positive natural killer (NK) cells mediated tumor cell killing (7). MSA has been reported to alter the expression of NKG2D ligands on cancer cells, thereby enhancing their recognition and elimination by NKG2D-expressing immune effector cells (8).

Immunosuppression is one of the major side effects in cancer patients treated with chemo and radiotherapy. The new frontier in anti-cancer treatment is the concept of stimulating endogenous immune response against tumor cells by removal of elements such as, influence of co-inhibitory receptors. Several strategies, like usage of immune checkpoint blockade with anti-PD-1/PD-L1 or CTLA-4 antibodies and cancer vaccines have been proposed in ovarian cancer to enhance immune cell response, especially of the T cells (9). The combination of chemo- and immunomodulatory treatment may benefit in both direct cytotoxic effects and the development of long-term antitumor immunity (10). These therapies might potentially enhance the susceptibility of tumor cells to T cell mediated tumor killing via different mechanisms.

In particular, suppression of regulatory T cells (Tregs) has been shown to aggravate the proliferation of implanted tumor cells in mice. Increased expression of programmed cell death ligand 1 (PDL1) on both tumor cells and dendritic cells (DCs), is known to cause T-cell exhaustion (11, 12). In ovarian cancer cell lines, treatment with various chemotherapeutic agents has been shown to upregulate PDL1 leading to suppression of antigen-specific T-cell function *in vitro*. In a mouse model of ovarian cancer, treatment with paclitaxel resulted in upregulated PDL1 expression. Treatment with a combination of paclitaxel and PDL1/PD-1 blockade resulted in longer survival compared with mice treated with paclitaxel alone (13). Moreover, factors produced in the tumor microenvironment have also been shown to cause exhaustion of T cells by up-regulation of PD-1 (14). Increased levels of vascular endothelial growth factor (VEGF) in the tumor microenvironment have for instance been shown to enhance the expression of PD-1 leading to CD8+ T cell exhaustion (15). VEGF can directly suppress activation of T cells isolated from ascites from ovarian cancer patients via the VEGF receptor-2 (16). Ovarian cancer cells have also been shown to act immunosuppressive as a mechanism of tumor immune escape and tolerance via increasing the infiltration and induction of Tregs, suppressing NK cell function, T cell activation, and T cell proliferation (17).

This study was primarily undertaken to investigate the effect of selenite and MSA on the immune system in terms of cell viability and activation at doses that are cytotoxic for the cancer cells. Furthermore, it also aimed to study the possible indirect immune modulatory mechanism of selenite and MSA through which they potentially exert by affecting tumor cells and/or the microenvironment. Based on the literature of the immunomodulatory effect of Se compounds, we thus hypothesize that selenite and MSA might alter the immune cells mediated tumor cytolytic activity.

## Materials and methods

### Sample collection, mononuclear cell isolation

Ascites was obtained from 4 ovarian cancer patients (International Federation of Gynecology and Obstetrics (FIGO) stage III or IV) at the Karolinska University Hospital, Solna, Sweden with written consent from donating patients and ethical approval granted by the Regional Ethical Review Board in Stockholm (Dnr 2013/2161-31). Heparin (5,000 IE/mL) was added upon collection during surgery. The ascites samples were centrifuged at 400 g for 7 min to remove excess liquid. Mononuclear cells were isolated from the samples by density gradient centrifugation using Lymphoprep (Fresenius Kabi) and centrifugation at 800 g for 20 min. The mononuclear cell fraction was washed twice in PBS (at 500 g for 10 min) and thereafter either cultured fresh or was frozen in complete 1640 RPMI medium (Thermo Fisher Scientific) containing 10% human AB serum (Karolinska University Hospital), 1 % PEST (100 U penicillin/mL and 100 μg streptomycin/mL, Thermo Fisher Scientific) and containing 10% DMSO (WAK-Chemie Medical GmbH). Frozen samples were stored at −192°C until usage.

Peripheral blood mononuclear cells (PBMCs) of healthy donors (*n* = 15) were isolated using density gradient separation (Lymphoprep-Lonza). NK cells (CD56 biotinylated) and T cells (CD45 RA, clone HI 100) were purified using MojoSort Streptavidin Nanobeads (BioLegend) by following manufacturer protocol. T cells were further FACS sorted in FACSVantage (BD Biosciences) using anti-human CD3 PE (Miltenyi Biotec).

### Cell culturing conditions and generation of conditioned media

The human ovarian cancer cell lines (A2780 and CP70), PBMCs as well as immune cells derived from ascites were maintained in RPMI 1640 media with ultraglutamine I (Lonza) supplemented with 10% fetal bovine serum (FBS) (GE Healthcare HYCLONE) and 2 mM glutamine (Gibco, Life Technologies); in 5% CO_2_ at 37°C. To study the induction of hypoxia, cells were treated with selenite or MSA for 4 h and quickly lysed in cytoskeletol buffer (10 mM PIPES, 300 mM NaCl, 1 mM EDTA, 300 mM sucrose, 1 mM MgCl_2_, 0.5% TritonX 100, Phosphatase inhibitor) supplemented with protease inhibitor cocktail (Roche) for protein extraction. Conditioned media was generated by culturing A2780 or CP70 cells as described above, exposing cells to 5 μM selenite (Sigma Aldrich) or MSA (Sigma Aldrich) for 24 h, where after the cell culture media was collected for further experiments. When indicated extra VEGF (PeproTech) (1 ng/ml) was added daily for 48 h where T cells were cultured in tumor conditioned media.

### Quantification of thiols

Free thiols in the culture medium were quantified using 300 μL of medium with final concentrations of 200 mM Tris-HCl (pH 8.0), 2 M guanidine hydrochloride, and 1 mM DTNB. Absorbance at 412 nm was measured using plate reader (SpectraMax 340PC, Molecular Devices).

### Cell viability

Cell viability was assessed in 96-well plates, either by crystal violet staining (Sigma-Aldrich), neutral red 40 μg/ml (Sigma -Aldrich), or by flow cytometry with 1:10 dilution of AnnexinV-FITC (BD Biosciences) and PI 5 μg/ml (Sigma Aldrich). The latter was analyzed on a BD FACS Callibur (BD Biosciences) and the data were analyzed using FlowJo V10 (BD Biosciences).

### Western blotting

40 μg of proteins were separated on a Bolt^TM^ 4–12% Bis-Tris Gel (Novex) and transferred to a nitrocellulose membrane using the iBlot Gel Transfer Device (Invitrogen). The membranes were then probed with rabbit monoclonal anti-human PDL1 (E1L3N, Cell signaling Technology), rabbit monoclonal anti-human HIF-1α (D2U3T, Cell signaling Technology)) and mouse monoclonal anti-human β-actin (A5441, Sigma- Aldrich). Incubation with primary antibody diluted in TBST containing 5% dry non-fat milk was done overnight at 4°C. Secondary antibodies (1:5,000 in TBST with 5% dry milk) were incubated for 1h at room temperature. Membranes were developed using the Amersham^TM^ ECL^TM^ Start Western Blotting Detection Reagent (GE Healthcare) and bands were visualized using the Bio-Rad Quantity One imaging system (Bio-Rad).

### Cytolytic assays

When indicated, recombinant human Interleukin-2 (IL-2) (PeproTech) was used for NK cell activation at a concentration of 1,000 IU/ml for 24 h prior to the lysis assay. T cells were stimulated with the human T cell-activator CD3/CD28 Dynabeads (Thermo Fischer Scientific) and 30 IU/mL IL-2 (PeproTech) for 96 h. Target cells were pre-labeled with fluorescent membrane staining PKH67 Green Fluorescent Cell Linker Mini Kit for General Cell Membrane Labeling (Sigma-Aldrich). Activated T cells and NK cells were co-incubated with target cells at different ratios, in a final volume of 420 μl for 3.5 h at 37°C and 5% CO_2_. At the end of the assay, cells were stained with PI (Sigma-Aldrich) to determine apoptosis by flow cytometry using BD FACS Calibur (BD Biosciences) and analyzed with FlowJo V10 (BD Biosciences).

### Multicolor flow cytometry

Multicolor flow cytometry was performed to identify T cells and NK cells in patient-derived ascites and analyze their expression of different surface activation markers. All antibodies were purchased from BD Biosciences and included FITC-conjugated anti-HLA-DR (G46-6), PE-conjugated anti-CD25 (M-A251), PE-conjugated anti-CD56 (MY31), PE-Cy7-conjugated anti-CD3 (SK7), Alexa700-conjugated anti-CD4 (RPA-T4), APC-Cy7-conjugated anti-CD69 (FN50) and V500-conjugated CD8 (RPA-T8). Cells were stained in a 96-well plate with titrated volumes of antibodies and incubated for 20 min at 4°C in the dark. After one wash with PBS, the cells were stained with 7AAD (BD Biosciences) for dead cell discrimination according to the manufacturer's instructions. After 10 min of incubation, PBS was added to all wells and the samples were acquired on BD FACSCanto I SORP (BD Biosciences) using FACSDiva V7 software (BD Biosciences). The data was analyzed using FlowJo V10 (BD Biosciences).

### Quantitative polymerase chain reaction (qPCR)

RNA was extracted using the RNeasy Plus Mini Kit (Qiagen) according to manufacturer's instructions and converted to cDNA using First strand cDNA synthesis Kit for RT-qPCR (Thermo Fischer Scientific). qPCR was performed in duplicates on 96-well plates using a PikoReal Real-Time PCR System (Thermo Fischer Scientific) and Luminaris Color HiGreen Master Mix (Thermo Fischer Scientific). Included primers were: HMOX (Forward- 5′-CCGACAGCATGCCCCAGGATT-3′ and Reverse-5′-GTCTCGGGTCACCTGGCCCTT-3′) and GCLM (Forward- 5′-CATTTACAGCCTTACTGGGAGG-3′ and Reverse-5′-ATGCAGTCAAATCTGGTGGCA-3′) (Integrated DNA Technologies), PDL1 (QuantiTect primer assay-QT00082775, Qiagen), MMP9, MMP13, MMP19 (Quantitech primer assay- QT00040040, QT00001764, QT00027286, Qiagen). Results were analyzed using the 2^−ΔΔC^_T_ method, and β-actin was used as endogenous control.

### Enzyme-linked immunosorbent assay (ELISA)

Tumor cells were harvested at 24 h and culture supernatant was analyzed for VEGF levels using an ELISA kit in accordance to the protocol provided by the manufacturer (Thermo Fischer Scientific and Mabtech). T cell culture supernatants were collected after 96 h of activation, and analyzed for Interferon gamma (IFNγ) and Granzyme B levels using an ELISA kit (Thermo Fischer Scientific) according to the manufacturer's instructions.

### Luminex assay

Media obtained from the co-culture of stimulated T cells and tumor pre-conditioned media were collected after 48 h. This media was then analyzed for cytokines using a Luminex Magpix-based assay (Luminex Corporation). 17 different soluble analytes (GM-CSF, sCD137, IFNγ,sFas, sFasL,Granzyme A, Granzyme B, IL-2, IL-4, IL-5, IL-6, IL-10, IL-13, MIP-1α, MIP-1β, TNF-α, and Perforin from the Human High Sensitivity T-Cell panel (HCD8MAG15K-17,Merck Millipore) were analyzed. This assay was performed at Clinical Immunology, Karolinska University Hospital, Sweden using manufacturer's instructions.

### Statistics

Statistical analyses were performed with GraphPad Prism (GraphPad). Student's *t*-test was applied on all the obtained data. Statistical significance was determined where *p*-value was < 0.05.

## Results

### Immune cells are more resistant to cytotoxic effects of selenite and MSA compared to the ovarian cancer cell lines A2780 and CP70

**Figure 1:**
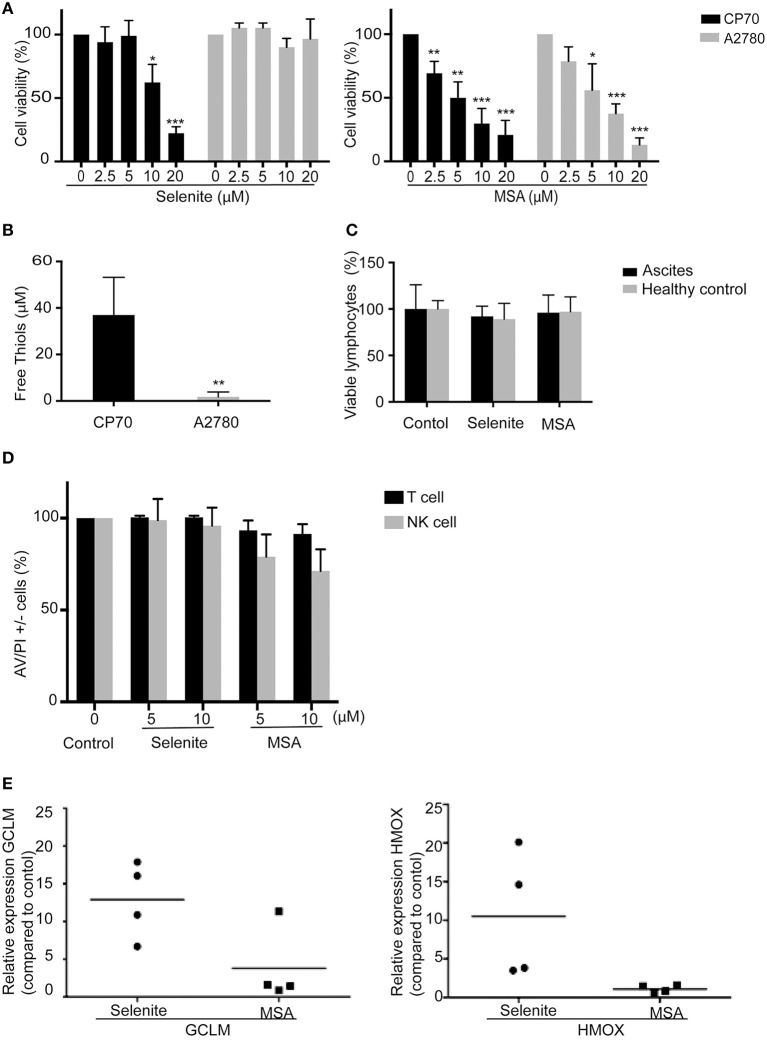
Immune cells are resistant to Selenite and MSA treatment at cytotoxic doses for ovarian cancer cells. Cell viability of tumor cells, peripheral blood mononuclear cells (PBMCs) and immune cells (NK and T cells) were assessed after treatment with selenite and MSA at different concentrations for 24 h. **(A)** Cell viability in % of tumor cells. **(B)** Free thiols in media obtained from tumor cells. **(C)** Viability % of lymphocytes obtained from healthy controls and ascites from ovarian cancer patients after treatment with selenite and MSA (10 μM) (*n* = 4). **(D)** % viability of immune cells (*n* = 4). **(E)** The mRNA levels of Nrf2 response genes (HMOX1 and GCLM) after treatment with selenite/MSA compared to control and relative to actin (*n* = 4). (bars indicate *SD*, ^*^p ≤ 0.05, ^**^p ≤ 0.01, ^***^p ≤ 0.001).

To assess the cytotoxicity of selenite and MSA, ovarian cancer cell lines and immune cells were treated for 24 h with varying concentration of selenite and MSA. Viability assay showed that A2780 cells were resistant to sodium selenite treatment (EC_50_ approximately at 70 μM, data not shown) whereas their cisplatin resistant counterpart, the CP70 cell line, were more sensitive to selenite (EC_50_ approximately between 10 and 12.5 μM) (Figure 1A). Moreover, in accordance with previous studies (18) sensitivity to selenite was coupled to the amount of free extracellular thiols in the media produced by the particular cell line, which in turn has been shown to be directly connected to the uptake of selenite (Figure 1B). However both cell lines where equally sensitive to MSA treatment (Figure 1A). Neither selenite nor MSA were toxic to lymphocytes from ovarian cancer patient ascites and healthy control at similar doses (Figure 1C). Even after sorting and assessing cell viability of NK and T cells, no significant cytotoxicity was observed with neither of the treatments (Figure 1D). Taken together these results suggest that NK cells and T cells are resistant to selenite and MSA at doses that are toxic to ovarian cancer cells.

### Resistance of immune cells to selenite is associated with upregulation of Nrf2 pathway.

Nuclear factor erythroid 2–related factor 2 (Nrf2) is a transcription factor induced by oxidative stress and upregulates antioxidant defense systems like Heme oxygenase-1 and NAD(P)H quinone oxidoreductase 1(Nqo1). To test if the immune cell resistance to selenite or MSA is a consequence of Nrf2 activation, a qPCR was performed to analyze the expression of genes regulated by Nrf2. Selenite induced a significant increase in the expression of GCLM and HMOX upon 24 h treatment of ascites-derived immune cells, however treatment with MSA did not trigger any Nrf2 response (Figure 1E). This result suggests that survival of immune cells upon treatment with selenite may be due to activation of Nrf2 target genes.

### Expression of lymphocyte activation markers upon pretreatment with selenite or MSA

**Figure 2:**
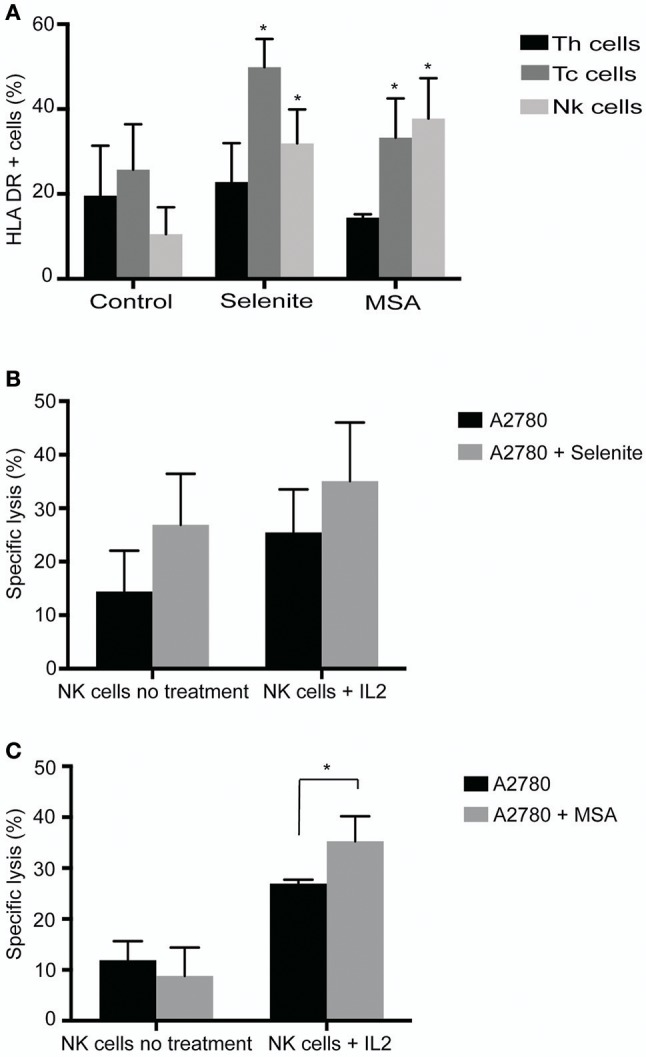
Selenite and MSA sensitizes tumor cells to NK cell mediated killing. Direct effects on immune cells (activation, shift in population) assessed after 24 h treatment with selenite and MSA. **(A)** HLA-DR expression on immune cell subsets following treatment with selenite and MSA (10 μM) (*n* = 3). **(B,C)** Specific killing of tumor cells monitored after co incubation with non-activated or overnight IL-2 activated NK cells with A2780 cells pretreated with selenite or MSA (10 μM) (*n* = 3). Columns represent mean specific killing (%); bar indicates *SD*. MSA and/or selenite treated samples were compared to the control (^*^p ≤ 0.05).

To test the direct effect of Se compounds on immune cell activation, immune cells isolated from ascites of ovarian cancer patients were treated with selenite or MSA for 24 h, and expression of activation markers HLA-DR, CD69, and CD25 were analyzed by flow cytometry. A significant increase in the expression of HLA-DR in NK cells (CD3–/CD56+) and CD8+ T cells (CD3+/CD4–CD8+) was observed upon treatment with selenite and MSA (Figure 2A). However, no significant alteration in the expression of activation markers CD69 and CD25 was observed by the treatments (data not shown). To further examine any alteration in the distribution of cellular populations in immune cell compartment of ovarian cancer patient ascites, we analyzed the proportion of CD4+ (T helper cells) and CD8+ T-cells (T cytotoxic cells). Treatment with selenite and MSA caused no significant shift in the proportion of these populations (data not shown).

### Pretreatment of ovarian cancer cell line A2780, with MSA enhances NK cell mediated tumor cell killing

To assess whether pretreatment of ovarian cancer cells with selenite or MSA results in increasing NK cell mediated tumor cell killing, A2780 cells were pretreated with selenite or MSA for 24 h. Pretreatment of A2780 cells with selenite for 24 h showed a trend of increased *in vitro* lysis efficiency of NK cells with approximately 10%, but not with MSA pretreatment (Figure 2B). Conventional NK cell activation with IL-2 (1,000 UI/ml, 24 h) prior to the lysis assay showed a trend for an even further enhanced effect with tumor cells pretreated with selenite even though not statistically significant. However, MSA pretreatment, in the IL-2 activated NK cells, had a significant increased lytic activity (Figure 2B). These results indicate that pretreatment of A2780 cells with MSA increases the lytic activity of NK cell.

### Pretreatment of ovarian cancer cell line CP70, with selenite or MSA decreases the expression of PDL1

**Figure 3:**
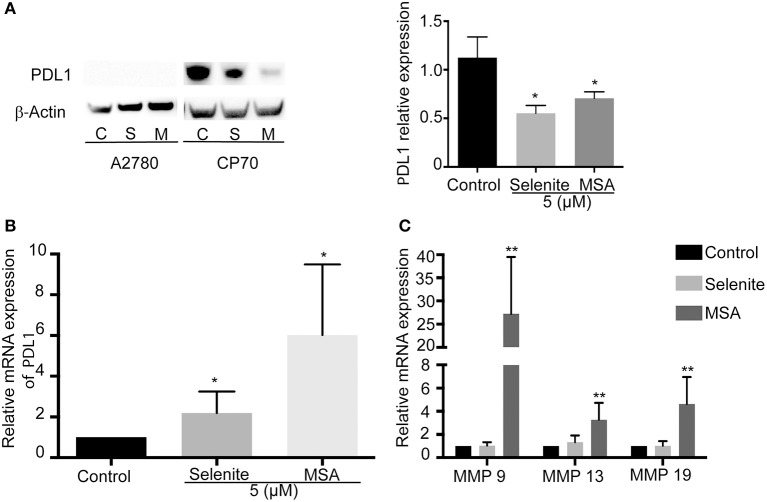
MSA decreases the protein levels of PDL1. **(A)** Western blot analysis of PDL1 (approximately 50 kDa) after treatment (5 μM) with selenite (S) or MSA (M) for 24 h. Graph shows quantification of PDL1 proteins normalized by β-actin. **(B)** Relative mRNA expression of PDL1 on treatment with selenite and MSA compared to control. **(C)** Relative mRNA expression of MMPs after selenite and MSA treatment compared to control. Columns represent mean expression levels (%); bar indicates *SD* (^*^p ≤ 0.05, ^**^p ≤ 0.01, ^***^p ≤ 0.001).

We next wanted to examine the effects of selenite and MSA on tumor cells in more detail. Using western blotting, PDL1 expression was measured on the ovarian cancer cells, and in accordance with previous studies where A2780 expressed low levels of PDL1 (19), PDL1 was here below detection limit, while the cisplatin resistant CP70 cell line showed very high basal levels of PDL1. The PDL1 protein expression was significantly decreased upon treatment with selenite and MSA (Figure 3A). The downregulation of PDL1 however did not occur at the transcriptional level as the mRNA expression of PDL1 was drastically increased upon MSA treatment (Figure 3B). To further understand the mechanism of PDL1 downregulation, the expression of matrix metalloproteineases (MMP9, MMP13, and MMP 19) were analyzed, as MMPs have been reported to degrade PDL1 (20). All the three MMP's were significantly upregulated in response to MSA (Figure 3C).

### T cell activation and increase in T-cell mediated killing of ovarian tumor cells by selenite and MSA

**Figure 4:**
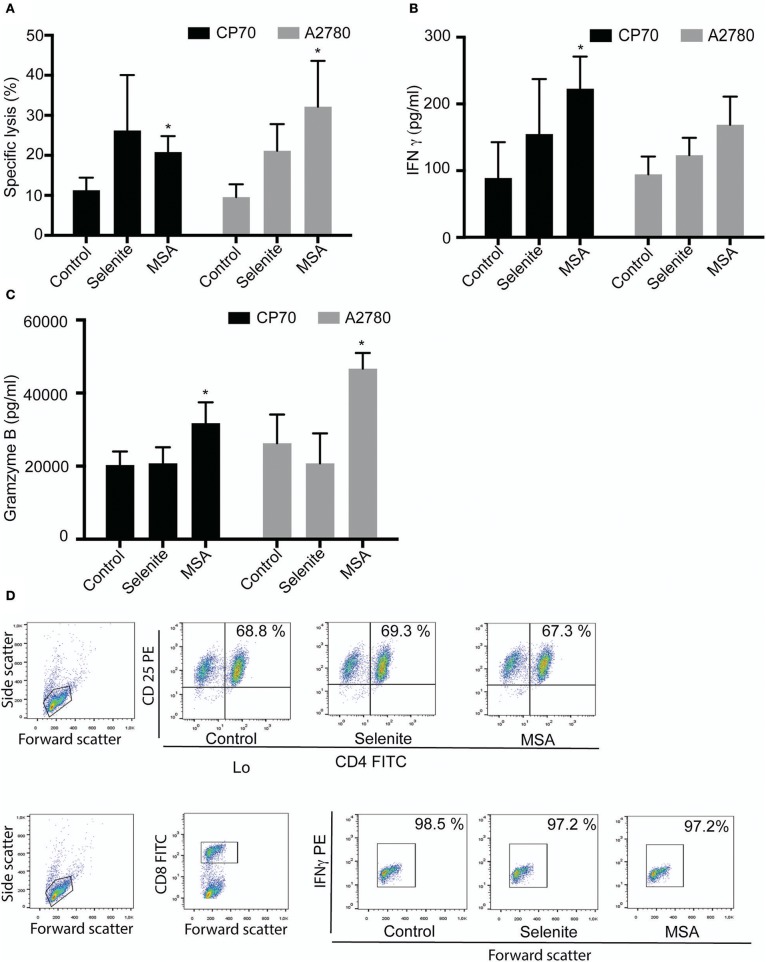
Tumor preconditioned media increases cytolytic activity of T cells. Activated T cells cultured in preconditioned media for 48 h followed by cytolytic measurements after co-incubation with new target cells. **(A)** Specific T cell killing of tumor cells (*n* = 3). Columns represent mean specific killing (%); bar indicates *SD*. **(B)** IFNγ levels in cell supernatant after incubation with T cells for 48 h (*n* = 3). **(C)** Granzyme B levels in cell supernatant after incubation with T cells for 48 h (*n* = 3). Columns represent mean IFNγ and Granzyme B levels (pg/ml); bar indicates *SD*. **(D)** Representative plots form one experiment showing shift in T cell population. MSA and/or selenite treated samples were compared to control (^*^p ≤ 0.05).

T cells were activated for four days and several soluble markers were used to confirm the activation (Supplementary Figure S1A). As both IFN γ and granzyme B increased above the upper detection limit in the Luminex analysis, separate ELISAs were performed for these markers, and both demonstrated a drastic increase after activation (Supplementary Figure S1B). To further evaluate if the preconditioned media from tumor cells pretreated with selenite or MSA for 24 h might have an additional effect on the lytic activity of T cells, activated T cells were incubated with preconditioned media for 48 h prior to incubation with fresh tumor cells for 3.5 h. An increased cytolytic activity of T cell cultured in preconditioned media from A2780 and CP70 cells treated with selenite or MSA was observed in both cell lines (Figure 4A). The enhanced lytic activity correlated well with the significantly elevated levels of IFNγ and granzyme B for the MSA treatment (Figures 4B,C). The incubation however did not alter the proportion of CD4+/CD8+ T cell subsets (Figure 4D). These results suggest that treatment with especially MSA not only alters the expression of PDL1 on the tumors, but also enhances the T cell mediated killing of tumor cells.

### MSA alters the levels of HIF-1α and VEGF, thereby increasing the T cell mediated tumor cell killing

**Figure 5:**
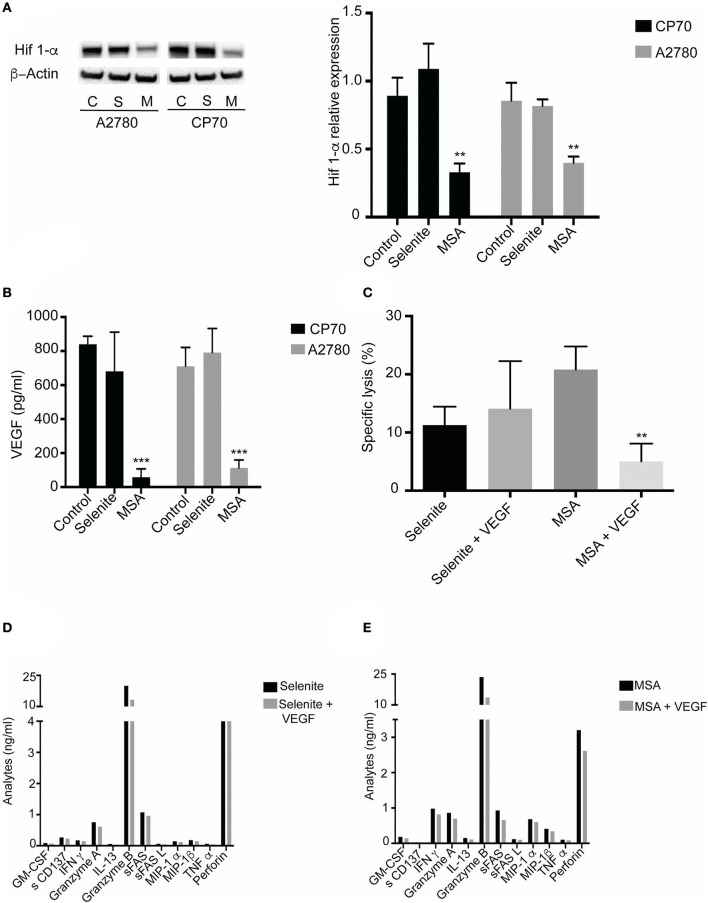
MSA treatment Decreases HIF**-**1α **(A)** Cells were treated (5 μM) with selenite and MSA under hypoxic condition for 4 h. Western blot analysis of HIF 1-α for CP70 and A2780 cells (approximately 120 kDa). Graph shows quantification of HIF 1-α protein normalized to β-actin. **(B)** Activated T cells cultured in preconditioned media with or without VEGF for 48 h. Killing was monitored after coincubation with CP70 cells. **(B)** VEGF levels from CP70 and A2780 treated with 5 μM selenite/MSA for 24 h (*n* = 3). Columns represent mean VEGF levels (pg/ml); bar indicates *SD*. **(C)** Specific T cell (with or without VEGF in culture) killing of CP70 cells (*n* = 3). **(D)** Representative experiment of T cell specific analytes from Luminex assay on addition of VEGF (^**^p ≤ 0.01, ^***^p ≤ 0.001).

To explore the effect by which T cells gain increased cytolytic activity after incubation in preconditioned media, HIF-1α levels were determined under hypoxic conditions. MSA clearly suppressed the protein levels of HIF-1α, whereas selenite treatment did not (Figure 5A). As HIF-1α is known to regulate the expression of VEGF, and VEGF in turn is known to inhibit T cell function, VEGF levels were measured in the preconditioned media. A more than 4-fold decrease in VEGF was observed in both cell lines treated with MSA. Again, no such effect was seen using selenite pretreatment (Figure 5B).

To assess if the enhanced cytolytic activity of the T cells was truly mediated via decreased levels of VEGF, VEGF was added to the preconditioned media in an attempt to block the observed effect. On addition of VEGF (1 ng/ml) to the T cells incubated with tumor preconditioned media (with selenite or MSA), the lytic activity exerted by the T cells was reversed compared to T cell incubated in preconditioned media from MSA (Figure 5C). Conversely, as selenite pretreatment did not affect the VEGF levels which remained high, additional VEGF did not alter the specific lytic activity in a significant way. The addition of VEGF to the preconditioned media from tumor cells was also followed by a decrease in cytokine production (Figures 5D,E). These results demonstrate that changes in VEGF levels by MSA contribute to the T cell mediated tumor cell killing.

### Effect of MSA on T cells

**Figure 6:**
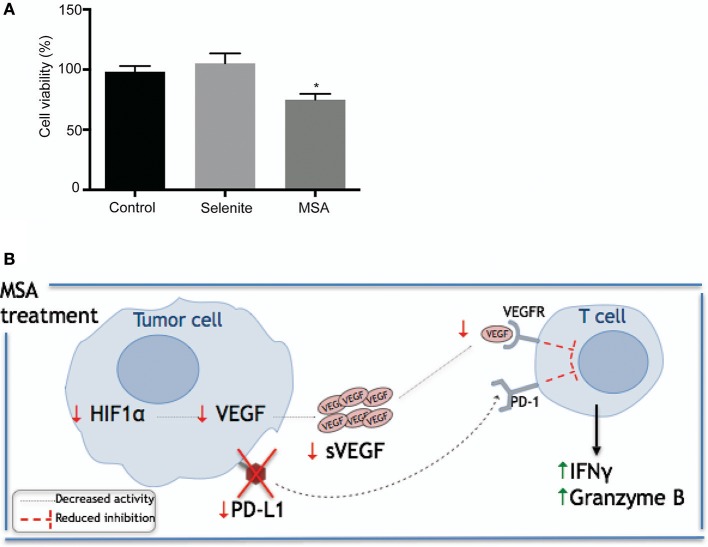
Schematic illustration of proposed mechanism by which MSA sensitizes tumor cells to T cell mediated killing. **(A)** Relative tumor cell killing by T cells in pretreated tumor cells. Activated T cells were cultured in a 96 well plate with the pretreated (with selenite and MSA 5 μM for 24 h) CP70 cells in media containing 1 μM treatment for 48 h. Tumor cell viability was measured using neutral red. Tumor cell killing represents percentage reduction in number of tumor cells, relative to control cultures without T cells. All experiments were performed with three biological replicates per group **(B)** Schematic representation of proposed mechanism for MSA. MSA and/or selenite treated samples were compared to the control (^*^p ≤ 0.05).

To further investigate the effect of the Se compounds on T cell cytotoxicity, stimulated T cells were cultured with pretreated (5 μM selenite or MSA for 24 h) tumor cells for 48 h. To maintain the low levels of PDL1, 1 μM treatment (both MSA and selenite) was added during the co-culturing for 48 h. There was an approximately 20% decrease in viability of tumor cells treated with MSA (Figure 6A). Figure 6B shows a graphical representation of the proposed mechanism for action of MSA in the tumor microenvironment.

## Discussion

Redox active compounds can readily form ROS and generate oxidative stress, which is generally believed to inhibit the immune system. This study demonstrates that the redox active selenium compounds selenite and MSA, at cytotoxic doses for ovarian cancer cells, does not inhibit or affect the viability of T cells and NK cells. To our knowledge, this is also the first time it has been demonstrated that enhancement of lytic activity by T cells on ovarian cancer cell lines can be mediated by MSA, through the inhibition of PDL1 and VEGF.

Our data suggests that selenite and MSA are selectively toxic to tumor cells, with the T cells and NK cells remaining unaffected by the treatments. Selenite is also known to be especially cytotoxic in drug resistant tumor cells (1). Concomitantly, in this study, a difference in the cytotoxicity of selenite was observed between A2780 and CP70 cell line where CP70 (Cisplatin resistant variant of A2780) was found to be more sensitive to selenite toxicity. Selenite's tumor specific killing (21–23), is mainly attributed to the redox cycling of selenide with oxygen generating superoxide, and eventually oxidative stress (24, 25). Selenite has however been reported to give rise to more systemic effects compared to organic selenium compounds like MSA. Xenograft mice models treated with MSA has for instance shown to reduce tumor growth without inducing any systemic toxicity or any other genotoxic side effects (26), and organic Se compounds have therefore been foreseen as more promising chemotherapeutic agents (2).

Selenite, known to induce intracellular oxidative stress, can trigger the Nrf2 transcriptional response, which is a central regulator of cellular defense against oxidative stress (27). Consistently, treatment of immune cells with selenite resulted in a strong upregulation of Nrf2 regulated genes, indicating a protective mechanism adopted by immune cells for survival. This might partly explain the survival of immune cells upon treatment with selenite. MSA has however been reported in several studies to act via redox modulatory effects that do not mainly cause oxidative stress to the cells (28). In line with these previous studies, MSA did not trigger any Nrf2 response, indicating that immune cell resistance to MSA is achieved by a different molecular mechanism, again illustrating the distinct effect encompassed by each separate selenium metabolite (29).

Several studies have described the correlation between selenium supplementation at supra-nutritional doses and immune responses, but studies on the effect of Se compounds at subtoxic doses on immune cell activation are scarce. One study showed indirect effect of selenite on NK cells, where selenite induced posttranscriptional inhibition of HLA-E and sensitized tumor cells to CD94/NKG2A positive NK cells (7). Herein, selenite showed the same trend, but did not significantly affect the ovarian cancer cell line. Similar results on NK cells have also been reported for MSA (8), and our data confirms this on ovarian cancer cells, as treatment of A2780 cells with MSA increases NK cell mediated lysis. This most likely occurs by the same mechanism as previously reported, with loss of the HLA-E antigen, since ovarian cancer cells also are known to possess DNAM-1 receptor (7), rendering the cells susceptible to NK cell mediated killing.

Sensitization of ovarian cancer cells to T cells via MSA could herein directly be linked to decreased levels of PDL1 in the CP70 cell line. Nevertheless, MSA does not seem to affect PDL1 at the transcriptional level as the mRNA expression increased dramatically. This further indicates that the effect might be at a posttranscriptional level. We recently demonstrated that MSA affect a large set of genes involved in the loss of attachment of cells to the extracellular matrix. Among the many genes identified, several of the matrix metalloproteineases (MMPs) were found to be significantly upregulated in response to MSA (28). Methylselenol, the active metabolite of MSA, was also identified by Zeng and co-workers to activate MMP-2 and MMP-9 (30). Consistently our results also show an upregulation of MMP9, MMP13 and MMP19 in response to MSA. MMPs have recently been shown to degrade PDL1 (20), thus the decreased protein levels of PDL1 after MSA treatment of CP70 cells found in the current study, may likely be explained via the induction of MMPs that subsequently degrade PDL1. A further study is warranted to confirm if the mechanism by which MSA downregulates PDL1 is via MMPs.

MSA treated bone metastatic mammary cancer cells have been reported to decrease VEGF levels (31). MSA have also been reported to inhibit HIF-1α expression and VEGF secretion in lymphoma cell lines and in prostate cancer cells (32). In both studies, authors connected the beneficial effects observed of VEGF inhibition to its angiogenic properties. However, in addition to its angiogenic properties (33, 34), VEGF also encompasses immunosuppressive effects (35), and there are many proposed mechanisms by which VEGF is used by tumors to achieve immunosuppression (36, 37). VEGF has been shown to suppress the activation of T cells from ascites from ovarian cancer patients via VEGF receptor type 2 (16). Secretion of VEGF by tumor cells has also been reported to induce endothelial cells to upregulate prostaglandin E2 (PGE2) which led to the suppression of T cell functions (38). Herein, our results show that incubation of activated T cells in tumor preconditioned media with selenite or MSA indeed enhanced the T cells mediated killing of CP70 and A2780 cells. This enhanced tumor killing activity of T cells is also supported by increased levels of granzyme B and IFNγ in the media. HIF-1α and VEGF were identified as key players with significantly lowered levels upon MSA treatment, and upon addition of VEGF, the lytic activity of T cells was reduced. The increase in lytic activity by MSA treatment could hence at least partially be explained via inhibition of the tumor secreted factor VEGF expression (37). Interestingly this observation was only seen for MSA and not selenite. MSA may therefore not only have an effect on angiogenesis via VEGF inhibition as stated in previous studies, but may also contribute to the upregulation of a tumor specific immune response, and may ultimately contribute in blocking the immune escape and progression of tumors.

Several studies have demonstrated that increased VEGF, HIF-lα, and PTEN expression in ovarian cancer cells can be used as predictors of metastasis and prognosis (39), and that increased plasma VEGF levels in ovarian cancer patients correlate to advanced tumor stage and a poor outcome (40). Compounds inhibiting VEGF may therefore be beneficial in ovarian cancer. From the present study we now know that selenite and MSA do not adversely affect the viability of immune cells at concentrations cytotoxic to tumor cells. Furthermore, we report that MSA decreases the levels of PDL1, HIF-1α, and VEGF in ovarian cancer cell lines and thereby renders the tumor cell sensitive to T cells, turning MSA into an interesting candidate for combinational treatments. MSA may contribute in tumor killing by two possible modes; (1) downregulating the expression of PDL1 thus limiting the activation inhibiting PD-1/PDL1 pathway, resulting in increased T cell activation and killing; (2) by decreasing HIF-1α expression which in turn leads to reduced VEGF levels in the tumor microenvironment. The reduction in VEGF levels will consequently increase the ability of T cells to kill tumor cells. Although further experiments are warranted to confirm these findings and animal models are required, these results suggest a potential strategy to use selenium compounds in anticancer treatment to boost immune functions.

## Author contributions

AF and DN: conception and design of the work; DN, ER, ND-A, AS, and PK: data collection; AF, DN, ER, ND-A, and PK: data analysis and interpretation; DN and AF: drafting the article; CK, PK, MU, JU, and ER: critical revision of the article; AF, MU, and JU: final approval of the version to be published.

### Conflict of interest statement

The authors declare that the research was conducted in the absence of any commercial or financial relationships that could be construed as a potential conflict of interest.

## Abbreviations

DC: dendritic cells
GCLM: Gamma-glutamylcysteine synthetase
HIF-1α: Hypoxic inducing factor 1α
HOX1: Heme oxygenase-1
INFγ: Interferon gamma
MMPs: matrix metalloproteineases
MSA: Methylseleninic acid
NK cells: natural killer cells
Nrf2: Nuclear factor erythroid 2–related factor 2
PBMC: peripheral blood mononuclear cells
PDL1: Programed cell death ligand 1
ROS: reactive oxygen species
Se: selenium
VEGF: vascular endothelial growth factor.
