# Supplementary material for: Methylseleninic Acid Sensitizes Ovarian Cancer Cells to T-Cell Mediated Killing by Decreasing PDL1 and VEGF Levels
Source: Front Oncol. 2018 Sep 28;8:407. doi: 10.3389/fonc.2018.00407 (PMC6172341; doi:10.3389/fonc.2018.00407)
Supplement: Supplementary file 1 [file Data_Sheet_1.docx]

***Supplementary Material***

**Methylseleninic acid sensitizes ovarian cancer cells to T-cell mediated killing by decreasing PDL1 and VEGF levels**

Deepika Nair*, Emelie Rådestad, Prajakta Khalkar, Nuria Diaz-Argelich, Axel Schröder, Charlotte Klynning, Johanna Ungerstedt, Michael Uhlin, Aristi P. Fernandes

Correspondence: Aristi Fernandes [aristi.fernandes@ki.se](mailto:aristi.fernandes@ki.se).

1. **Supplementary Data**

Supplementary data includes one figure.

1. **Supplementary Figures:**

**

**

**Supplementary Figure 1: Analysis of T cell activation**

A) T cells were isolated and stimulated with anti-CD3/CD28 for 96h, and confirmation for activation of T cells was performed using Luminex assay. # represents the values that had to be extrapolated from the standard curve and BD represents values below detection levels. B) ELISA for IFN γ and Granzyme B. Columns represent mean analytes levels; bar indicates SD. (*p ≤ 0.05, **p ≤ 0.01, ***p ≤ 0.001)
